# Intracranial Tumours and the Occurrence of Papillœdema

**Published:** 1923-03

**Authors:** A. E. Iles

**Affiliations:** Ophthalmic Surgeon to the Bristol General Hospital; Assistant Surgeon to the Bristol Eye Hospital


### INTRACRANIAL TUMOURS AND THE OCCURRENCE OF PAPILLŒDEMA.

BY

A. E. ILES, O.B.E., M.B. Lond., F.R.C.S.,

*Ophthalmic Surgeon to the Bristol General Hospital; Assistant Surgeon to the Bristol Eye Hospital.*

---

THE presence of papillœdema (optic neuritis) in cases of cerebral tumour or abscess is of such great diagnostic value that a consideration of the factors which cause this sign to be present or absent may be of interest. I have recently had under my observation six patients in whom the existence of cerebral tumour or abscess was confirmed either by operation or necropsy.

The sites of the tumour or abscess were as follows :—

Three of the tumours were in the frontal region.

One abscess in the optic thalamus.

One tumour in the occipital lobe.

One tumour in the pons.

CASE I.—D. P., æt. 19, was admitted to the Bristol General Hospital suffering from increasing loss of vision and facial palsy.

*On examination.*—There was facial paralysis on the left side, conjugate deviation of the eyes to the right, left pupil did not react to light. Vision, right, perception of light; eft, nil. The fundi showed, right, definite papillœdema with hemorrhages and white patches; the left, marked consecutive trophy.

*Operation.*—From the above signs the cranium was opened on the left side, but the tumour was found to be irremovable.

*Post-mortem.*—The tumour was found to be a rapidly-growing gliosarcoma in the pons on the left side in the region of the sixth nerve nucleus.

CASE 2.—Mrs. C., æt. 28, had suffered from severe and intractable headache following a motor accident, and presented other signs and symptoms indicative of a cerebral lesion. Immediately subsequent to the accident she suffered from diplopia ; this, however, proved to be transient.

*Vision.*—Fair in each eye, the left worse than the right. She could not be corrected to full vision.

No lesion of ocular muscles ; pupils reacted equally to light and accommodation.

*Fields of Vision.*—The right inferior quadrant of each field was defective. Small objects could not be seen. There was, however, a relative scotoma for larger objects in which a two-shilling piece could be recognised as " something," but no idea could be formed as to its nature.

Light thrown upon the defective area was followed by reaction of the pupils.

*Discs.*—Both showed marked swelling, the left more in amount than the right, hemorrhages and white patches being present in both.

The fields of vision and the presence of a relative scotoma localised the tumour in the cortex of the left cuneus.

*Operation.*—A gliosarcoma was found involving the cortex of the left cuneus.

CASE 3.—J. M., æt. 8. This girl had been in fair health until a fortnight before admission, save when she had suffered from bilateral middle ear disease with intermittent discharge from her ears ever since.

She first became dull and heavy on November 14th, was admitted on November 28th, when she was found to be irritable with a vacant stare—photophobia, and general tenderness and stiffness of her neck muscles. Urine normal on admission.

*Course.*—She soon began to suffer from short bouts of unconsciousness accompanied by signs of cerebral irritation, restlessness, etc., with attacks of severe vomiting. Kernig's sign gradually developed. One month after admission 0.3 per cent. albumen appeared in the urine. Three days after the appearance of albuminuria she began to suffer from short convulsions lasting about one minute, with twitching of the face and limbs ; these ceased, however, after lumbar puncture. One week later she had bleeding from the rectum, kidney and mucous membrane of the mouth ; at this time much pus was found in the urine.

*Fundi.*—Moderate bilateral papillœdema, with hemorrhages on left disc, none on right ; no evidence of œdema of hemorrhages or exudation in the retina. A later examination showed the same characteristics ; at this time, however, slight weakness in movement of the eyes to the right was present.

The left eye was the one in which the papillœdema had first appeared.

*Cerebro-spinal fluid.*—November 30th, less than 10 cells in field. December 22nd, 40 cells, of which 90 per cent. were lymphocytes. December 31st, more than 500 cells to the field. The presence of the large number of cells in the cerebro-spinal fluid, together with the rapid onset of papillœdema, pointed to either an abscess discharging into a ventricle, or tuberculous meningitis (acute).

*Post-mortem.*

1. Acute ascending nephritis with sloughs of bladder.

2. Small extradural cholesteatomatous mass found at junction of squamous and petrous temporal, not in connection with either the middle ear or lateral sinus.

3. Excess of clear fluid in the ventricles.

4. An abscess one-third square inch in left optic thalamus surrounded by a larger hemorrhagic zone, with engorgement of the overlying ependyma. Microscopically this abscess was shown to be discharging into the lateral ventricle.

Case 4.—P. B., æt. 19. In 1920 this patient had a motor-cycle accident, striking his right frontal region.

In the latter end of 1921 he suffered from headache. On December, 1921, he had a fit with twitching of the left side of the body, accompanied by severe headache and vomiting.

This fit was succeeded by many others, still accompanied by headache and vomiting. Lumbar puncture relieved this condition for a time, causing freedom from fits for four months. After this time had elapsed they recurred, with headaches which were more severe in character. I saw him shortly after this second attack, and found bilateral papillœdema, the left disc being the more swollen. (It is interesting that I had seen this patient before this accident, and found his fundi normal, a small degree of hypermetropic astigmatism only being present.) The fits and localising signs, however, pointed to a right-sided lesion. The diagnosis of a tumour in the right pre-Rolandic area was made. At the operation a large endothelioma, $2\frac{1}{2}$ in. by 2 in., was found growing in the right frontal region. This was successfully removed.

Case 5.—M. A., æt. 27. This patient was first seen by me in out-patients for headache. No lesion of the fundus was then seen, and no refractive error found. She had, however, in the right frontal region an irregularity of the skull, and the left side of the body was markedly wasted ; this wasting she stated had been present since she was seven years of age. Three months later I saw her again ; she had had in the meantime an attack of "influenza" with headache and slight vomiting. Vision then 6/18 each eye, and papillœdema of each disc about 3 D., the two sides were equal. She was told that she should be admitted, but she did not come back for six weeks, when she was quite blind. Some time after admission her right side began to waste rapidly, and she gradually became weaker and died, the discs having passed into consecutive atrophy.

*Post-mortem.*—The following condition was found : a tumour in the right frontal region replacing most of the frontal lobe, compressing the head of the caudate nucleus and pushing it backwards, it also extended inwards forming a depression on the mesial aspect of left frontal lobe.

CASE 6.—J. M., æt. 67, admitted to the Bristol General Hospital stuporous and complaining of pain in the head but without any vomiting. Discs were found to have very deep physiological cups in each, were slightly red. No swelling of either disc was present, no hemorrhages or exudation found. There was a history of a previous prostatectomy.

*Post-mortem.*—A tumour, $1\frac{1}{2}$ in. by 1 in., found in left frontal lobe and extending outwards. No sign of tumour on brain surface, mesial or lateral. The tumour was found to be a secondary carcinoma, origin from prostate.

### SUMMARY.

Five of the cases showed papillœdema, and the sixth did not.

Cases 1, 2, 4 and 5 were of comparatively long duration. Cases 3 and 6 were of comparatively short duration.

Case 1.—The discs in this case were markedly different, the right side still showing papillœdema, whilst the left (that on the side of the lesion) showed consecutive atrophy.

The presence of facial palsy and conjugate deviation of the eyes pointed to a lesion of the left side of the pons.

Case 2.—The discs were in the advanced stage, white spots having appeared on the upper nasal edges of the discs.

The presence of a positive scotoma of a part of the field of vision (homonymous in type) with a marked relative scotoma showed that the lesion was situated in the region of the left cuneus.

Case 3.—Hemorrhages present in one disc only, and that on the same side as the lesion.

Bilateral papillœdema with hemorrhages on left disc only. This condition showed that the swelling in the left disc had been present before the right.

This patient had had bilateral ear disease. Examination showed that the aural condition was satisfactory ; also no signs or symptoms pointing to involvement of the temporo-sphenoidal or cerebellar regions were found. Although albuminuria was present, the appearance of the fundi were not characteristic of renal disease.

The rapid increase in the number of leucocytes in the cerebro-spinal fluid pointed, however, to abscess. From this the suggestions were made that it was either an abscess discharging into one of the ventricles, or tuberculous meningitis with considerable hydrops venticuli.

The case is a particularly instructive one ; the presence of such a small abscess giving rise to marked intra-ocular signs, together with the fact that the abscess was discharging across into the ventricles, is worthy of record.

Case 4.—Unequal swelling of the discs, the disc showing the greater amount of swelling being on the side opposed to the lesion.

The left disc in this case was the one which showed the greater amount of swelling ; therefore, according to Sir Victor Horsley, the tumour should have been on the left side. Can the fact that the ocular signs were contra-lateral be explained ?

Leonard Hill, in his *Physiology and Pathology of the Cerebral Circulation*, points out that the presence of the falx cerebri is a factor which interferes with the ability of the cerebro-spinal fluid (if under pressure) to exert that pressure equally in all directions.

In this particular case the tumour was growing from the meninges in the right frontal region, and therefore in its growth tended to press the frontal lobe backwards and to the left (part of this increased pressure to the left would be taken up by the falx).

The optic nerve before it enters the optic foramen lies in close relationship with the frontal lobe, therefore increases of pressure on the frontal lobe would be transmitted uniformly through the thickness of the brain substance to the optic nerve. The right frontal lobe would therefore by its pressure obstruct the flow of the cerebro-spinal fluid along the right optic nerve sheath, thus accounting for the appearance of papillœdema in the left eye at an earlier date than the right. This theory is in direct opposition to that of Schieck, since if the latter were correct the presence of obstruction on the right side would have resulted in earlier papillœdema on that side.

Case 5.—The papillœdema in each eye was equal in extent.

In this case the tumour was of very large size, it had grown to such an extent that pressure was directly transmitted by the falx (which had been pushed to the left) to the left frontal lobe, it had also extended very far back and was pressing upon the caudate nucleus.

This case therefore differs from Case 4 in that the pressure was more directly distributed to the base and also to the opposite side.

Case 6.—No papillœdema present.

In this case the tumour was in the actual brain substance, and was extending in an outward direction. There was no sign of direct pressure from the tumour upon the base; the growing pressure was also directed away from the mesial aspect of the brain, whereas in the two preceding tumours of the frontal lobe the growing pressure was directed backwards, downwards and to the left.

Sir Victor Horsley in 1910 claimed that the eye in which papillœdema first became manifest was on the same side as the cerebral lesion, and brought forward a series of cases to support his contention. With Marcus Gunn he described the order in which changes appear in the disc. Shortly they are as follows :—

*First sign.*—Appearance of swelling of the upper and inner edge of the affected sign.

*Second.*—Differences in amount of swelling of the discs.

*Third.*—Appearance of hemorrhages on the disc.

*Fourth.*—Appearance of white spots on upper nasal edge.

*Fifth.*—Consecutive atrophy.

Mott points out that the cerebral veins are surrounded by an intercerebral prolongation of the subarachnoid.

Mamourian and Smith claim that the sorption of cerebro-spinal fluid takes place chiefly through the superior cerebral veins.

The choroidal plexus secretes the cerebro-spinal fluid.

Mamourian and Smith contend on these grounds that increased intracranial pressure is due to deficient absorption of the cerebro-spinal fluid by the cerebral veins, and any blockage, particularly of the venous sinuses, is responsible for the rise in pressure and consequent driving of the fluid forward into the subarachnoid space around the optic nerve. This, in their view, explains the rapid appearance of papillœdema in cases where the sinuses are affected, *e.g.* middle-ear disease and complications, anæmia with longitudinal sinus thrombosis ; also in subtentorial swellings and those in the neighbourhood of the corpora quadrigemina.

Leslie Paton has stated that the ipsolaterality of papillœdema is not at all constant. My cases tend to show that tumours in the frontal lobe, although of large extent, may give widely varying appearances in the optic discs. They also seem to show that the direction and rate in which the tumour grows are definite factors governing the appearance of papillœdema, and determining the side in which the swelling of the disc first makes itself manifest.

The three other cases, situated in the optic thalamus, occipital cortex and pons, showed definite ipsolaterality; the tumour in the pontine area showing the greatest difference between the two discs.

### Progress of the Medical Sciences.

### PSYCHIATRY.

